# Comparative Transcriptomic Profiling in Two Widely Used Human Cell Lines Delineates Their Shared and Distinct Patterns of Interferon Responses

**DOI:** 10.3390/biology15161418

**Published:** 2026-08-18

**Authors:** Jiayao Jiang, Liangliang Zhang, Qianyi Yang, Shuai Chen, Ming-An Sun

**Affiliations:** 1 Institute of Comparative Medicine, College of Veterinary Medicine, Yangzhou University, Yangzhou 225009, China; dx120220184@stu.yzu.edu.cn (J.J.); zll05101534@163.com (L.Z.); mx120241081@stu.yzu.edu.cn (Q.Y.); 2 Joint International Research Laboratory of Important Animal Infectious Diseases and Zoonoses of Jiangsu Higher Education Institutions, Yangzhou 225009, China; 3 Jiangsu Co-Innovation Center for Prevention and Control of Important Animal Infectious Diseases and Zoonosis, Joint International Research Laboratory of Agriculture and Agri-Product Safety of Ministry of Education of China, Yangzhou 225009, China

**Keywords:** interferon response, interferon-stimulated gene, RNA-seq, cell specificity, HeLa, HEK293T

## Abstract

Interferons (IFNs) are a family of cytokines which serve as the first line of defense against pathogen infections while also exerting critical immunomodulatory roles. IFNs can induce hundreds of IFN-stimulated genes (ISGs) with cell-specificity, yet a high-resolution comparison of time-serial ISG induction across cell types is lacking. By using RNA sequencing, we conducted a comparative transcriptomic profiling during time-serial IFN-γ stimulation for up to 24 h in HeLa and HEK293T cells, the two most widely used immortalized human cell lines. We uncovered remarkably stronger IFN responses in HeLa cells, regarding the global transcriptomic dynamics, the number of induced ISGs, and the level of ISG expression. Both cell lines share a core set of ISGs associated with canonical JAK-STAT signaling, yet HeLa uniquely activates additional inflammatory and adaptive immunity-related pathways. Despite the much weaker IFN response in HEK293T cells, we also identified a few HEK293T-specific ISGs, including several with crucial immune-related functions. Notably, transposable elements—including many adjacent to ISGs—are also highly up-regulated in HeLa cells, implying their potential links to ISG induction. Collectively, this study provides a high-resolution temporal atlas of IFN-γ-stimulated transcriptomic dynamics in HeLa and HEK293T cells, revealing the shared core module and cell-specific patterns of their interferon responses.

## Simple Summary

Interferons (IFNs) are critical for pathogen resistance and immune modulation. While IFN response occurs in many cell types, a high-resolution comparison across cell types is lacking. By using RNA sequencing, this study conducted a time-serial charting of the transcriptomic responses in two widely used immortalized human cell lines, HeLa and HEK293T, during IFN-γ stimulation. We uncover stronger IFN response in HeLa cells, regarding the global transcriptomic dynamics, the number of IFN-stimulated genes (ISGs), and the level of ISG expression. Both cell lines share a core set of ISGs associated with canonical JAK-STAT signaling, yet HeLa uniquely activates additional inflammatory and adaptive immunity-related pathways. Despite the much weaker IFN response in HEK293T cells, we still identified a few HEK293T-specific ISGs, including several crucial immune-related genes. Notably, transposable elements—including many adjacent to ISGs—are also highly up-regulated in HeLa cells, implying their potential links to ISG induction. Collectively, this study provides a temporal atlas of IFN-γ stimulated transcriptomic dynamics in HeLa and HEK293T cells, which reveals the shared core module and cell-specific patterns of their interferon responses.

## 1. Introduction

Interferons (IFNs) constitute a family of cytokines that serve as the first line of defense against pathogen infections while also exerting broad immunomodulatory functions [1,2,3]. There are three families of IFNs: type I (IFN-I) that primarily contains IFN-α/β, type II (IFN-II) with IFN-γ, and type III (IFN-III) with IFN-λ [2]. Unlike type I and III IFNs, which show potent antiviral activities, the type II IFN-γ is distinguished by its central role in cell-mediated immunity against intracellular pathogens, as well as immunoregulatory roles [4,5,6]. IFN-γ signals through a heterodimeric receptor composed of IFNGR1 and IFNGR2 that activates the Janus kinases JAK1 and JAK2 and subsequently phosphorylates the core transcription factor (TF) named Signal transducer and activator of transcription 1 (STAT1). The phosphorylated STAT1 then forms homodimers, termed γ-activated factor (GAF), that bind to γ-activated sequences (GASs) to activate the expression of numerous interferon-stimulated genes (ISGs) [7,8]. The temporal ISG dynamics of IFN-γ signaling is complex: early responses are dominated by direct STAT1 targets (e.g., IRF1, SOCS1, and CIITA), whereas later waves involve secondary transcriptional cascades driven by newly synthesized TFs (e.g., STAT1 and IRF1), which can amplify and diversify the initial signal [9,10]. Mechanistically, despite the fact that the canonical JAK-STAT pathway is essential for ISG activation, the full IFN-induced transcriptional outputs and biological effects also require cooperation with non-canonical signaling cascades, such as the p38 mitogen-activated protein kinase (MAPK) pathway [8,11]. These pathways cooperatively fine-tune the activation of ISGs, thereby contributing to the diverse outcomes of IFN-γ stimulation, which range from antiviral protection and immunoregulation to macrophage activation [8,12].

While most human cells can express IFN receptors and respond to IFN stimulation, the IFN-stimulated gene responses differ remarkably across cell types [3,13]. Different cell types can exhibit marked heterogeneity in ISG induction kinetics and magnitude, reflecting differences in baseline chromatin accessibility, receptor abundance, and expression of signaling regulators [7,14]. However, a comprehensive time-resolved comparative study of IFN-γ-induced transcriptional responses across cell types is still lacking. HeLa (cervical cancer cells) and HEK293T (embryonic kidney cells) are among the two most widely used immortalized human cell lines, which have also been employed to study innate immune responses, including the activation of the IFN pathway. For example, numerous studies about the innate immune responses to different types of IFNs (e.g., IFN-α/β/γ) involve experiments by using either HeLa [15,16,17,18,19,20,21,22,23] or HEK293T [24,25,26,27,28,29,30] as in vitro models. Of note, relevant studies also include many of the earliest ones that contribute to the characterization of the IFN pathway [31,32,33,34,35]. Therefore, by direct comparison between these two cell lines, it is promising to uncover the cell-specificity of IFN response across cell types.

Apart from the activation of ISGs, numerous studies also link transposable elements (TEs), mobile DNA elements that constitute about half of the human genome, to the IFN-induced transcriptional responses [36,37,38]. There are two major classes of TEs, namely retrotransposons (class I) and DNA transposons (class II). Retrotransposons are further classified into long terminal repeat (LTR) elements, long interspersed nuclear elements (LINEs), and short interspersed nuclear elements (SINEs) [39]. Of note, most LTR elements are originated from ancient retroviral infection of host germline; hence, they are also termed as endogenous retroviruses (ERVs). Increasing evidence indicates that TEs, particularly LTRs/ERVs, participate extensively in IFN responses, either by forming cis-regulatory elements (e.g., promoters and enhancers) or producing RNA/protein molecules [16,40,41,42,43,44]. Importantly, TE expression is dynamically regulated during IFN stimulation and/or viral infection, and TE-derived double-stranded RNA molecules (dsRNA) can also enhance the activation of IFN pathway via “viral mimicry”, suggesting the feed-forward loop wherein TEs may further amplify the IFN response [38,42,45,46]. These findings position TEs as an important component of the IFN response network and hint at the links between TEs and cell-specific IFN responses.

Here, we conducted a comparative transcriptomic analysis of HeLa and HEK293T cells during time-serial IFN-γ stimulation by RNA sequencing (RNA-seq), which reveals the shared core module and cell-specific patterns of the interferon responses between cell lines.

## 2. Materials and Methods

### 2.1. Cell Culture

HeLa cells were gifted by Qiuchun Li’s lab (Yangzhou University, Yangzhou, China), and HEK293T cells were gifted by Yanhua Li’s lab (Yangzhou University, Yangzhou, China). Cells were authenticated by STR profiling and confirmed to be free of mycoplasma contamination by using qPCR with the primers targeting 16S rRNA gene (forward: GGGAGCAAACACGATAGATACCCT; reverse: TGCACCATCTGTCACTCTGTTAACCTC). Both cell lines were cultured in high-glucose Dulbecco’s Modified Eagle Medium (DMEM; Gibco, New York, NY, USA), supplemented with 10% fetal bovine serum (FBS; Sigma Aldrich, St. Louis, MO, USA) and 1% penicillin–streptomycin (P/S; Gibco, New York, NY, USA), in a humidified incubator at 37 °C with 5% CO_2_. When cell confluence reached 80–90%, subculture was performed at a split ratio of 1:3 to 1:5. Culture medium was changed every 2–3 days.

### 2.2. Interferon Stimulation

HEK293T and HeLa cells were seeded into 6-well plates at appropriate densities to reach approximately 70% confluence at the time of stimulation. The culture medium was then removed, and the cells were gently washed with PBS. Fresh complete medium was added, followed by the addition of recombinant human IFN-γ (PBL, 11500) at the concentration of 1000 U/mL, following previous studies [40,41,47]. For both cell lines, samples were collected at 0 h (untreated control), 30 min, 1 h, 3 h, 6 h, 12 h, and 24 h post-stimulation. Of note, an additional 15 min time point was included for HeLa cells to examine if any genes can respond after so short a time period of IFN-γ stimulation. All stimulations were performed in two biological replicates, each derived from separate cell cultures and IFN-γ-stimulation experiments.

### 2.3. RNA-Seq

Total RNA was extracted from HeLa and HEK293T cells using the FastPure Cell/Tissue Total RNA Isolation Kit V2 (Vazyme, RC112-01, Nanjing, China). mRNA was purified with poly-T magnetic beads, fragmented, and reverse-transcribed into cDNA. After PCR amplification, the RNA-seq libraries were sequenced as 150 bp paired-end reads on the DNBSEQ T7 platform (BGI, Shenzhen, China).

### 2.4. Gene Expression Analysis

Raw reads were trimmed with Trim Galore v0.6.4 and then aligned to human reference genome (GRCh38) using STAR v2.7.3 [48]. Gene-level read counts were obtained with featureCounts from Subread v2.0.0 [49]. Differentially expressed genes (DEGs) were identified by comparing each group of IFN-stimulated samples against the unstimulated controls using DESeq2 v1.30.1 [50] with the cutoff: FDR < 0.05, |log_2_FoldChange| > 1. Principal component analysis (PCA) was performed on rlog (regularized log)-transformed read counts, with the top 1000 (when integrating all the data for HeLa and HEK293T samples) or 100 (when analyzing the data for HeLa and HEK293T cells separately) most variable genes selected for analysis and visualization by using the plotPCA function of DESeq2. Transcripts per million (TPM) values were calculated with RSEM v1.3.2 [51]. Representative genomic tracks were visualized using IGV v2.13.1 [52]. Of note, ISGs are technically defined as the genes up-regulated after IFN-γ stimulation, which may differ from the broader sets of IFN-responsive genes in curated databases that involve multiple IFNs across diverse cell types. Mfuzz clustering analysis of ISGs was performed by using Mfuzz v3.23 [53], with calculated TPM values.

### 2.5. TE Expression Analysis

Subfamily-level TE expression analysis was performed using TEtranscripts v2.2.3 [54]. RNA-seq reads were aligned to human reference genome (GRCh38) using STAR v2.7.3 [48], with the parameters recommended by TEtranscripts: --outFilterMultimapNmax 100, --winAnchorMultimapNmax 100. This setting allows the reporting of sufficient number of multi-mapped reads. TEtranscripts then applies expectation-maximization (EM) algorithm to assign multi-mapped reads to the most possible TE subfamilies. Differentially expressed TE subfamilies were identified with TEtranscripts by invoking DESeq2 v1.30.1 [50] with the cutoff: FDR < 0.05, |log_2_FoldChange| > 1.

Locus-level TE expression analysis was performed using SQuIRE v0.9.9.92 [55] with default settings. SQuIRE presents multiple tools, including Fetch for reference annotation file preparation, Map for read alignment, Count for expression quantification, and Call for differential expression analysis. Multi-mapped reads are assigned to individual genomic loci by employing an iterative read-reassignment strategy. Differentially expressed TE loci were filtered with the cutoff: FDR < 0.05, |log_2_FoldChange| > 1.

### 2.6. Functional Enrichment Analysis

Gene Ontology (GO) and Kyoto Encyclopedia of Genes and Genomes (KEGG) enrichment analyses were performed using DAVID and clusterProfiler v4.10.1 [56,57]. As defaulted, we used all human genes as the background for enrichment analysis. Family-wise error rate was corrected with the Benjamini–Hochberg method, and enriched terms with adjusted *p*-value (p.adjust) < 0.05 were considered significant.

Gene set enrichment analysis (GSEA) was performed with the FGSEA v1.39.4 [58], which is a fast implementation of the original GSEA method [59]. Genes were ranked in descending order by the log2foldChange obtained from differential expression analysis. Gene sets were obtained from the org.Hs.eg.db package v3.23.1. Gene set permutation was performed with 1000 permutations, as defaulted. Family-wise error rate was corrected with the Benjamini–Hochberg method, and enriched gene sets with p.adjust < 0.05 were considered significant.

### 2.7. Reference Genome and Annotations

The reference genome and gene annotation for human (GRCh38) were retrieved from the Ensembl database [60]. TE annotations for the corresponding human genome were obtained from the UCSC Genome Browser [61]. To assess the lineage-specificity of TE subfamilies, we retrieved relevant annotations from Dfam database [62].

### 2.8. Statistical Analysis and Visualization

All statistical analyses were performed using the R statistical programming language (v4.6.0). The heatmaps of gene expression data were generated using the R package pheatmap v1.0.13. Representative RNA-seq tracks were visualized using Integrative Genomics Viewer (IGV) v2.13.1 [52].

## 3. Results

### 3.1. Distinct Global Transcriptomic Dynamics in HeLa and HEK293T Cells by IFN-γ Stimulation

**Figure 1:**
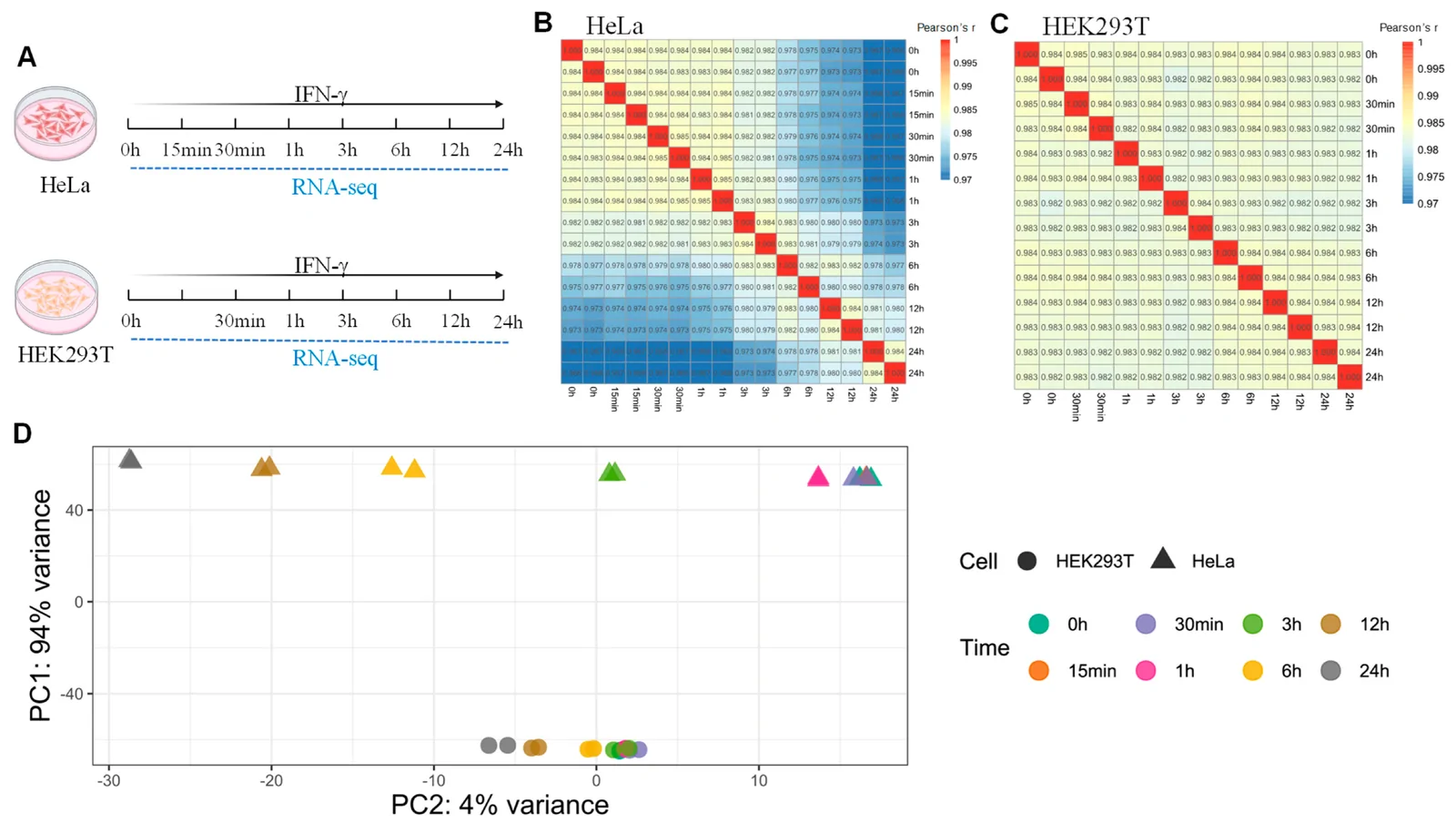
Global transcriptomic dynamics in HeLa and HEK293T cells during IFN-γ stimulation. (**A**) Scheme of the experimental procedure. HeLa and HEK293T cells were subjected to time-serial IFN-γ stimulation, and then collected for RNA-seq. (**B**,**C**) Heatmaps show the correlation of different RNA-seq samples during IFN-γ stimulation for HeLa and HEK293T cells, respectively. The color gradients represent the Pearson’s *r*, as calculated from normalized gene-level read counts. (**D**) PCA plot shows the global transcriptomic dynamics of HeLa and HEK293T cells in response to IFN-γ stimulation. The top 1000 most variable genes are used for PCA analysis. Here, PC2 (but not PC1) is highlighted as the *x*-axis, because it largely reflects the IFN-induced changes, as focused on by this study. Of note, the PCA plots with the data for HeLa and HEK293T cells analyzed separately are also provided as Figure S2.

To compare the IFN-stimulated transcriptomic responses in different cell types, we conducted RNA-seq for HeLa and HEK293T cells with time-serial stimulation (ranging from 0 to 24 h) by recombinant human IFN-γ (Figure 1A). Of note, an additional 15 min time point was set for HeLa cells to examine if any genes can be induced so early. Each sample has two biological replicates that were cultured and stimulated independently, and the unstimulated samples (0 h) were used as controls. To avoid potential batch effects, all experiments for both cell lines were performed simultaneously, and subsequent RNA-seq library preparation and high-throughput sequencing were also performed in the same batch. Routine quality control statistics, Pearson’s correlation, and PCA results confirmed the good quality of the generated RNA-seq data and the high consistency between replicated samples (Figure 1B,C, Figures S1 and S2; Table S1). In HeLa cells, we observed high correlations between unstimulated samples and the samples stimulated for less than 1 h, whereas the samples treated for 3–24 h showed obviously lower correlations with the unstimulated samples (Figure 1B), indicating extensive transcriptomic alterations after 1 h of IFN-γ stimulation. In contrast, the correlations between unstimulated and IFN-stimulated samples (even for those stimulated for as long as 24 h) for HEK293T cells show much higher correlations than those for HeLa cells (Figure 1C), indicating limited global transcriptomic fluctuation in HEK293T cells.

To better assess the global transcriptomic dynamics across cell types, we further inspected the PCA result which integrates all the samples of both HeLa and HEK293T cells (Figure 1D). As demonstrated by the PCA plot, the PC1 (vertical direction) mainly represents the differences between cell types, while PC2 (horizontal direction) represents the IFN-induced alterations. Given that HeLa and HEK293T are from different tissues, it is expectable that their inherited differences beyond the IFN-induced changes. We focus only on PC2, since IFN response is the focus of this study. Overall, the IFN-induced dynamics of the transcriptomic data show a similar trend in HeLa and HEK293T cells, yet the degree of changes differs remarkably (Figure 1D), in line with the much stronger transcriptomic alterations in HeLa cells, as indicated by correlation analysis (Figure 1B,C). Specifically, the 0–1 h samples in HeLa cells cluster together, and they are separated from the samples stimulated for longer than 3 h. While the overall trend in HEK293T cells resembles that of HeLa cells, their transcriptomes begin to disperse from the unstimulated samples only after 6 h of IFN-γ stimulation, and the distances between stimulated and unstimulated groups are considerably smaller than those in HeLa cells (Figure 1D). Together, IFN-γ stimulation induces global transcriptomic dynamics in both HeLa and HEK293T cells, yet the degree of their transcriptomic alterations differs remarkably, underscoring their distinct global transcriptomic responses upon IFN-γ stimulation.

### 3.2. More Genes Exhibit IFN-Regulated Expression in HeLa than in HEK293T Cells

**Figure 2:**
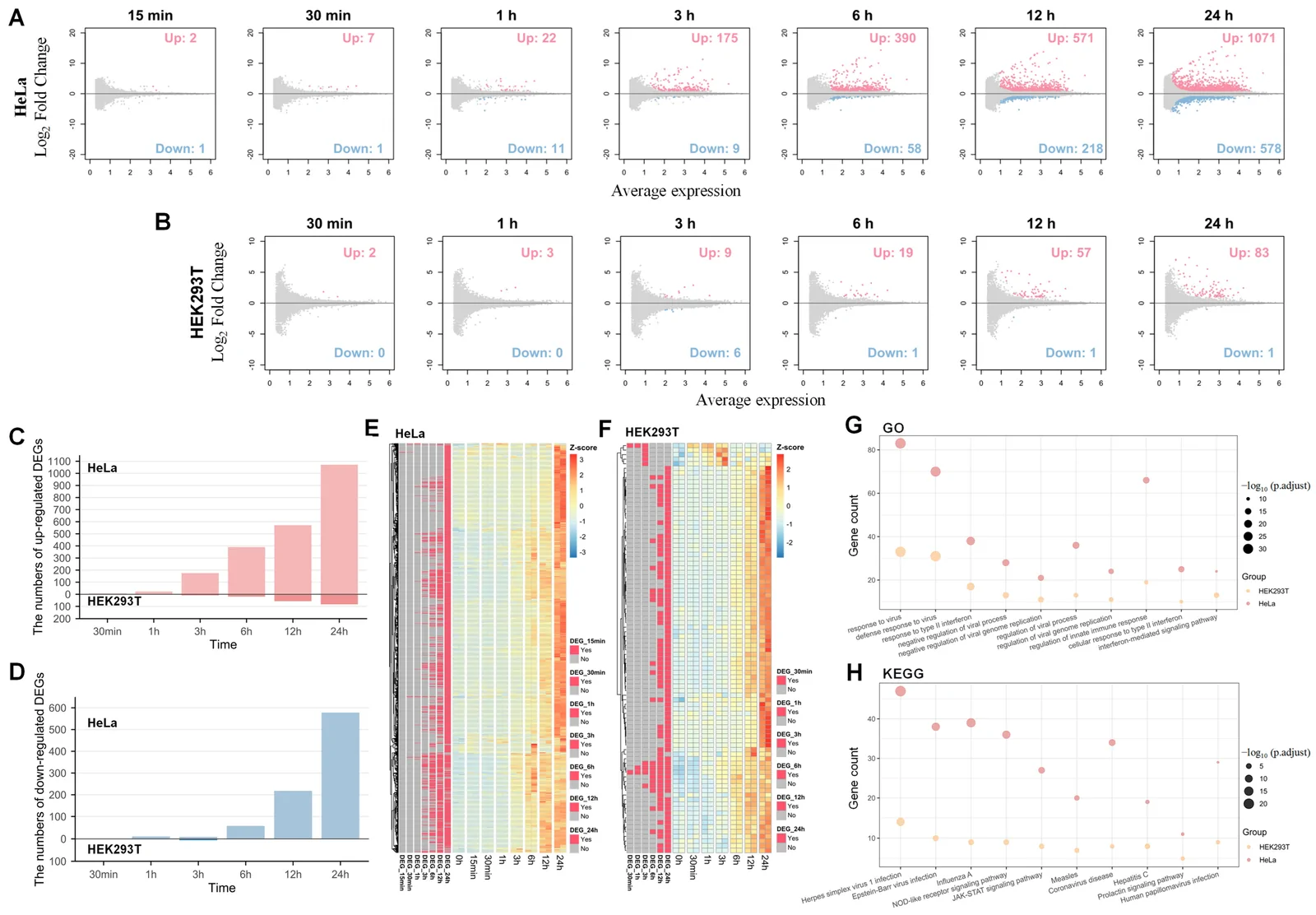
Characterization of the IFN-regulated genes in HeLa and HEK293T cells. (**A**,**B**) MA plots show the differential gene expression during IFN-γ stimulation in HeLa and HEK293T cells. The unstimulated group (0 h) was used as the control for differential expression analysis. The significantly up- or down-regulated genes are highlighted in red and blue, respectively. (**C**,**D**) The numbers of up- and down-regulated DEGs during IFN-γ stimulation in HeLa and HEK293T cells are shown. (**E**,**F**) Heatmaps show the temporal expression profiles of the ISGs in HeLa and HEK293T cells. The color gradients represent the row Z-score calculated from normalized gene-level read counts. (**G**,**H**) GO and KEGG enrichment analysis results for the ISGs identified in HeLa and HEK293T cells are presented.

To systematically compare the IFN responses in HeLa and HEK293T cells, we identified DEGs at each time point during IFN-γ stimulation. We identified numerous up- or down-regulated genes whose numbers increased progressively during IFN-γ stimulation, and the number of DEGs was substantially larger in HeLa cells than in HEK293T cells (Figure 2A,B and Table S2). In HeLa cells, the number of up-regulated genes rose sharply from 7 at 30 min to 1071 at 24 h, and down-regulated genes also increased from 1 to 578 during IFN-γ stimulation (Figure 2C,D). In contrast, only 83 up-regulated genes were identified in HEK293T cells even at 24 h, and down-regulated genes remained at extremely low numbers at all time points (Figure 2C,D). Notably, the two genes, *EGR1* and *FOS*, are induced at as early as 15 min in HeLa cells (Table S2), both belonging to immediate early genes, which can respond rapidly upon stimulation or stress [63]. We further visualized the expression profiles of all the ISGs, technically defined as the up-regulated genes during IFN-γ stimulation. Our result demonstrates that they were massively induced from 3 h onward and maintained at high expression level through 6–24 h in HeLa cells, whereas the level of ISG induction was only weak or moderate in HEK293T cells (Figure 2E,F). The remarkable differences in the ISGs between HeLa and HEK293T cells is in line with the patterns, as reflected by global transcriptomic analysis (Figure 1B–D).

To achieve more functional insights, we performed GO and KEGG enrichment analyses for the DEGs identified in each cell line (Figure 2G,H). We demonstrate that the up-regulated genes for both HeLa and HEK293T cells were highly associated with IFN-related pathways (e.g., response to virus, response to type II interferon, and JAK-STAT signaling pathway), with HeLa cells exhibiting higher numbers of genes across these pathways, as is expected given that they have more DEGs. Moreover, GSEA analysis also reveals the activation of the interferon-mediated signaling pathway in both cell lines (Figures S3 and S4). We also inspected the down-regulated genes in HeLa cells and found that they are associated with biological processes such as extracellular matrix organization, xenobiotic metabolic process, and regulation of anion transport (Figure S5), indicating that these fundamental pathways are suppressed during IFN-γ stimulation. Collectively, the core IFN pathway is activated by IFN-γ stimulation in both cell types, yet the breadth and intensity of ISG activation in HeLa cells are beyond those in HEK293T cells, thus highlighting the cell-specific transcriptional responses during IFN-γ stimulation.

### 3.3. TE Expression Correlates with the ISG Induction During IFN-γ Stimulation

**Figure 3:**
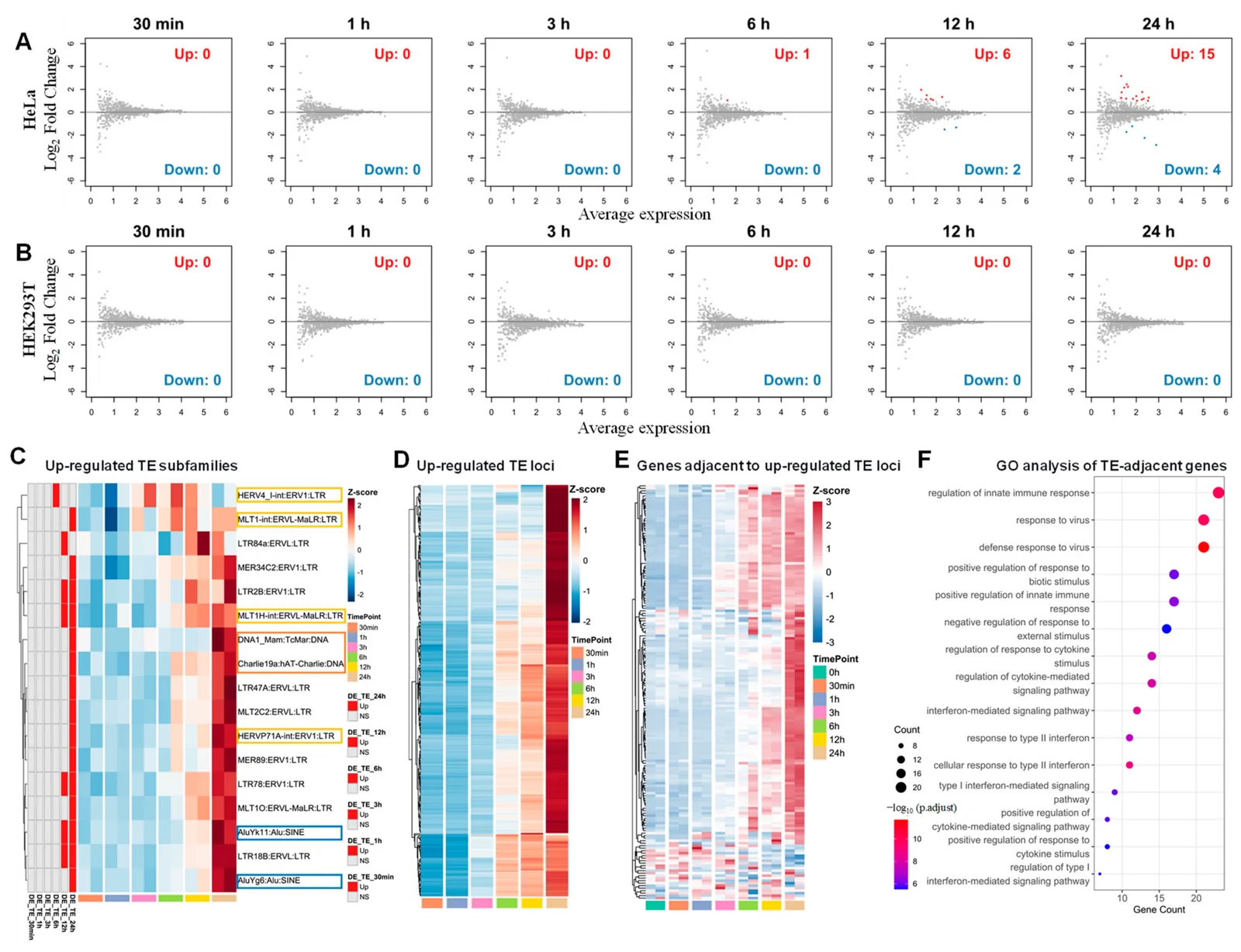
Altered expression of TEs and adjacent genes during IFN-γ stimulation in HeLa and HEK293T cells. (**A**,**B**) Expression changes of TEs at subfamily-level in HeLa and HEK293T cells during IFN-γ stimulation. (**C**–**E**) Expression profiles of the TE subfamilies, TE elements, and the genes adjacent to the up-regulated TE loci during IFN-γ stimulation in HeLa cells. The color gradients represent the row Z-score, as calculated from normalized read counts for TEs. (**F**) GO analysis of the genes adjacent to the up-regulated TE loci during IFN-γ stimulation in HeLa cells.

Intrigued by previous studies on the involvement of TEs in innate immune response [36,42], we further examined the expression profile of TEs in HeLa and HEK293T cells during IFN-γ stimulation. We first analyzed TE expression at the subfamily level, aiming to determine the specific TE subfamilies showing altered expression during IFN-γ stimulation (Figure 3A,B). In HeLa cells, one up-regulated TE subfamily (HERV4_I-int) is identified at 6 h, which belongs to primate-specific ERVs [62]. Then, the number of up-regulated subfamilies increased to six at 12 h and 15 at 24 h (Figure 3A). In contrast, only a small number of down-regulated TE subfamilies were detected at 12 h (*n* = 2) and 24 h (*n* = 4). Hence, we only focus on the up-regulated TE subfamilies. After merging the results of up-regulated TEs across all time points, we obtained a total of 17 subfamilies, with the majority (76.5%, *n* = 13) belonging to ERVs (Figure 3C), suggesting that ERVs constitute the predominant group of TEs responsive to IFN-γ stimulation. Notably, they involve both young ERV subfamilies specific to primates (e.g., HERVP71A-int and HERV4_I-int) and ancient ones shared by other mammals (e.g., MLT1H-int and MLT1-int). Apart from ERVs, a few subfamilies belonging to DNA transposons (i.e., Charlie19a and DNA1_Mam) or SINEs (i.e., AluYk11 and AluYg6) also show up-regulated expression. We further found that these 17 TE subfamilies are usually induced after 6 h in HeLa cells (Figure 3C). In contrast, no differentially expressed TE subfamily was identified in HEK293T cells at any time point (Figure 3B), correlating with the lower degree of gene induction in HEK293T cells during IFN-γ stimulation.

Subsequently, we performed locus-level expression analysis of TEs, aiming to identify the specific TE loci that are up-regulated by IFN-γ stimulation and characterize their links to ISGs. Notably, locus-level TE quantification remains technically challenging due to the highly repetitive nature of TE sequences, and SQuIRE handles multi-mapped reads by employing an iterative read reassignment strategy [55]. Consistent with the subfamily-level results, the expression changes of TEs in HEK293T cells are also much weaker than those in HeLa cells (Figures S6 and S7); hence, we only focus on HeLa cells. We identified a total of 351 unique up-regulated TEs in HeLa cells, with their expression predominantly induced at 6–24 h (Figure 3D). We further identified the genes adjacent to up-regulated TE loci and found that many of them also exhibit a clear temporal up-regulation during IFN-γ stimulation (Figure 3E and Table S3). GO and KEGG enrichment analysis further revealed that these genes were significantly enriched in immune-related processes, such as innate immune response, defense response to virus, and NOD-like receptor signaling (Figure 3F and Figure S8), which resemble the results for the identified ISGs (Figure 2G,H). Importantly, these TE-adjacent genes also include multiple ISGs with crucial roles, such as *STAT1/2*, *IRF1*, *OAS1/3*, *GBP1/2*, and *STING1*. Collectively, the IFN-induced expression of TEs shows cell-specific patterns and is associated with the induction of adjacent ISGs. However, whether the activated TEs contribute to the cell-specific induction of adjacent ISGs is still unclear and needs further clarification.

### 3.4. Comparative Analysis Uncovers the Shared and Cell-Specific ISGs in HeLa and HEK293T Cells

**Figure 4:**
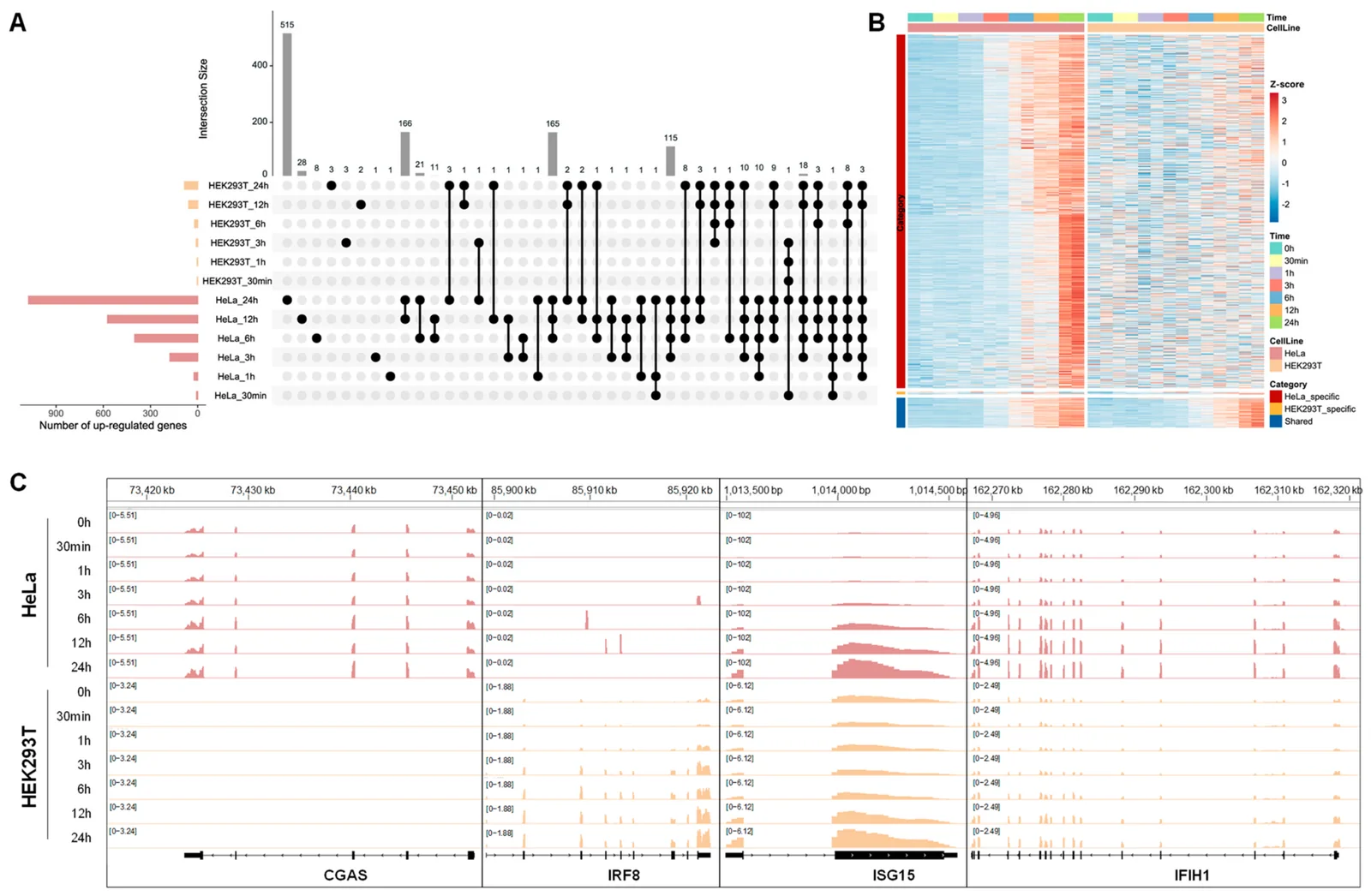
Comparison of the shared and cell-specific ISGs in HeLa and HEK293T cells. (**A**) UpSet plot shows overlaps among the ISGs identified in HeLa and HEK293T cells. (**B**) Heatmap shows the expression profiles of the shared and cell-specific ISGs for HeLa and HEK293T cells. To better visualize the expression dynamics during IFN-γ stimulation, the data for HeLa and HEK293T cells are normalized separately for visualization. We also present the result with the data for both cell lines normalized together (Figure S10), which is useful for the direct comparison of gene expression level across cell lines. The color gradients represent the row Z-score, as calculated from normalized gene-level read counts. (**C**) IGV tracks show the expression profiles of representative ISGs shared between or specific to HeLa and HEK293T cells.

To better dissect the cell-specificity of the IFN-induced transcriptional response, we further examined the ISGs shared between or specific to HeLa and HEK293T cells (Table S4). Through UpSet visualization, we first compared the lists of ISGs at each time point of IFN-γ stimulation in these two cell lines (Figure 4A). The most prominent intersection pattern was the ISGs unique to HeLa at 24 h (*n* = 515). In addition, there are also large number of genes shared by 12 and 24 h (*n* = 166), 6–24 h (*n* = 165), or 3–24 h (*n* = 115) in HeLa cells (Figure 4A), indicating that ISG activation displayed a highly ordered temporal pattern which usually persists throughout the late phase. In contrast, the unique ISGs at any individual time point in HEK293T are rare, and the constant activation of ISGs across time points is also rare (Figure 4A). Comparison across cell types also uncovers many shared ISGs, particularly between 24 h of HEK293T and several time points (e.g., *n* = 8 for 6–24 h, and *n* = 10 for 3–24 h) in HeLa cells (Figure 4A), yet the shared ISGs are relatively limited due to the small number of ISGs identified in HEK293T cells.

We further compared the pooled lists of ISGs for each cell line (*n* = 1127 for HeLa; *n* = 90 for HEK293T) and found that the majority (87.8%, *n* = 79) of the ISGs for HEK293T are also identified in HeLa cells (Figure S9A). Meanwhile, there are also 12.2% (*n* = 11) of ISGs in HEK293T cells that are absent from HeLa cells (Figure S9A), indicating the HEK293T-specific activation of these genes. Of note, only one out of the six IFN-repressed genes in HEK293T cells is also identified in HeLa cells (Figure S9B), indicating that the IFN-induced repression of genes in these two cell lines is highly divergent. We then compared the expression profiles of the shared and cell-specific ISGs in each cell line during IFN-γ stimulation, with the HeLa and HEK293T data normalized separately or together to highlight the dynamic patterns within or across cell lines, respectively (Figure 4B and Figure S10). As expected, HeLa-specific ISGs exhibit strong induction in HeLa cells while remaining at a low level in HEK293T cells, and shared ISGs are activated in both cell lines. Notably, even the shared ISGs are usually induced to remarkably higher level in HeLa relative to HEK293T cells, as demonstrated by the integrated expression heatmap and Mfuzz clustering plots (Figures S10 and S11). We then manually inspected each group of ISGs and confirmed the clear cell-specificity of HeLa or HEK293T-specific ISGs. For the shared ISGs, they cover many core genes of the IFN pathway, including TFs like *STAT1*, *STAT2*, *IRF1*, and *IRF9*, as well as immune effectors like *ISG15*, *OAS3*, and *IFIT2/3* (Figure 4C and Figure S12; Table S4). We also noticed many important ISGs specific for HeLa cells, such as *CGAS*, *GAP1*, *CXCL10/11*, *OAS1*, *IFIT1*, *MX2*, *STAT3*, and *SOCS1*, and multiple GBP or TRIM proteins (Figure 4C and Figure S12; Table S4). Despite the low number of ISGs for HEK293T, we also observed several critical immune genes, such as *IRF7*/*8* and *IFITM2*, that are specifically induced in HEK293T cells (Figure 4C and Figure S12; Table S4). Collectively, it seems that both cell lines share the core IFN-responsive module, yet HeLa achieves a higher amplification of the downstream transcriptional response relative to HEK293T cells.

### 3.5. Functional Inference of Shared and Cell-Specific ISGs in HeLa and HEK293T Cells

**Figure 5:**
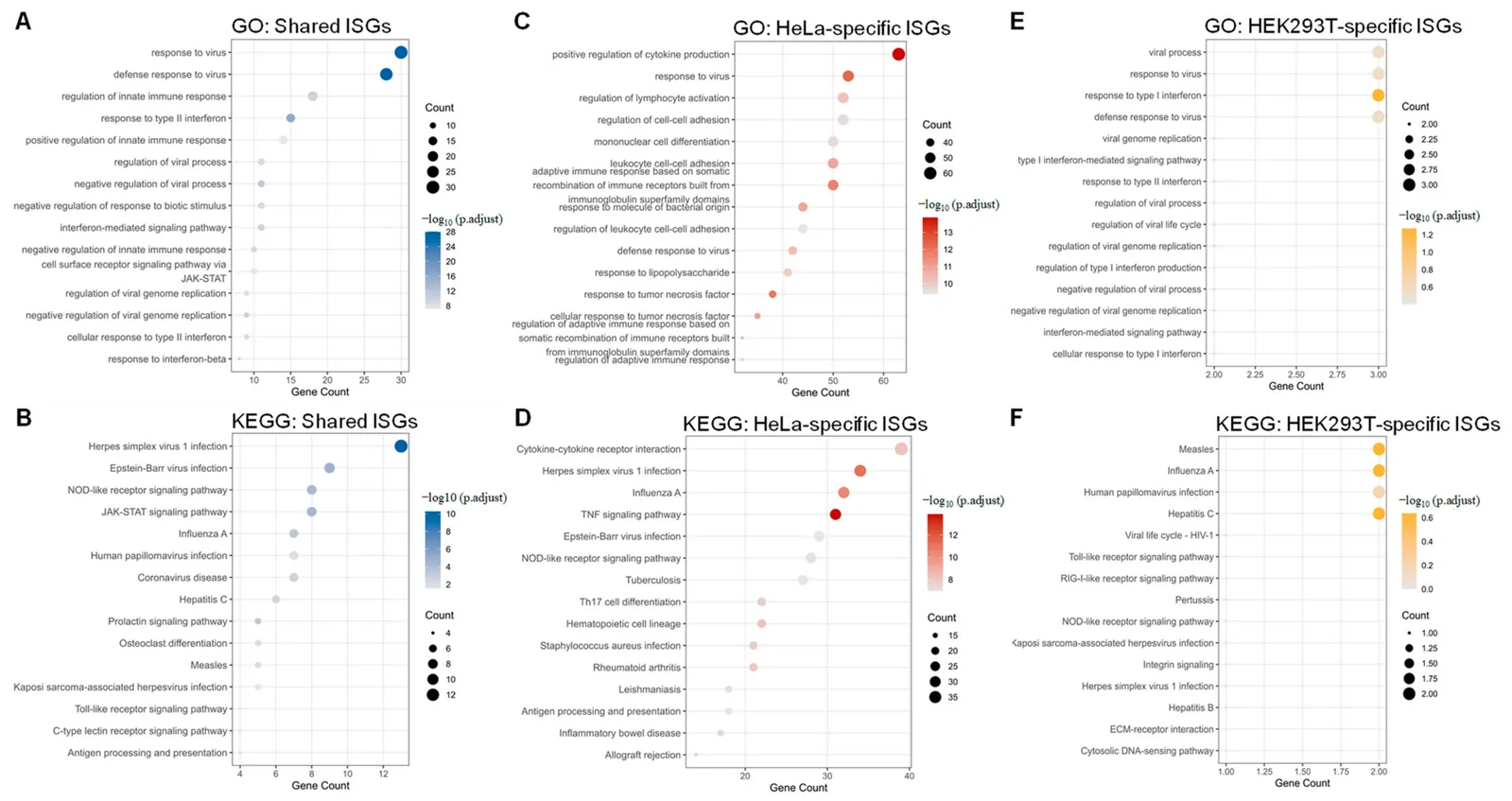
Functional relevance of the shared and cell-specific ISGs in HeLa and HEK293T cells. (**A**,**B**) GO and KEGG enrichment results for the ISGs shared by HeLa and HEK293T cells. (**C**,**D**) GO and KEGG enrichment results for the HeLa-specific ISGs. (**E**,**F**) GO and KEGG enrichment results for HEK293T-specific ISGs.

After uncovering the shared and cell-specific ISGs between HeLa and HEK293T cells, we further dissected the functional relevance of different groups of ISGs through GO and KEGG enrichment analyses. Overall, the shared ISGs are enriched with multiple pathways that are tightly associated with core IFN-γ response, such as response to virus, regulation of innate immune response, response to type II interferon, and JAK-STAT signaling pathway (Figure 5A,B). Therefore, the shared ISGs are tightly associated with the core IFN-responsive module, in line with the results of manual inspection of ISGs (Figure 4C and Figure S12). While HeLa-specific ISGs are also associated with IFN response and innate immunity, they are also enriched with many alternative pathways, such as those related to cytokine production, cell–cell adhesion, mononuclear cell differentiation, TNF signaling, immune receptors, and adaptive immune response (Figure 5C,D), indicating that the IFN-γ response in HeLa cells extends to cytokine signal amplification, immune cell differentiation, and adaptive immune regulation. Despite the limited number of HEK293T-specific ISGs precluding effective GO/KEGG enrichment analysis, we still observed their moderate association with pathways related to interferon response, and defense response to virus (Figure 5E,F), indicating that HEK293T cells could also exhibit cell-specific activation of a few immune or antiviral effectors.

## 4. Discussion

The IFN responses can differ remarkably across cell types, yet a high-resolution transcriptomic comparison of IFN-stimulated responses across cell types is missing. This study comprehensively compared the transcriptomic dynamics during time-serial IFN-γ stimulation (up to 24 h) between HeLa and HEK293T cells, which are among the most widely used human cell lines that have also been adopted by many studies focusing on IFN response [15,16,17,18,19,20,21,22,23,24,25,26,27,28,29,30]. Through in-depth comparative analysis, this study uncovers many shared and cell-specific features of the IFN responses, as discussed below.

A major finding of this study is the remarkably different magnitudes of gene responses between HeLa and HEK293T cells during IFN-γ stimulation, as reflected by the global transcriptomic alterations, the speed of IFN response, the numbers of induced ISGs, and the level of ISG expression. Overall, much more IFN-regulated genes are identified in HeLa relative to HEK293T cells, and the degree of expression alterations is also more evident in HeLa cells. During IFN-γ stimulation, HeLa cells not only activated the core ISG set but also induced many cell-specific ISGs, while most of the genes induced in HEK293T cells are shared by HeLa cells. Notably, HeLa-specific ISGs also include *SOCS1*, a crucial JAK-STAT pathway suppressor that contributes to IFN desensitization [8,64,65], indicating the establishment of a more complex feedback regulatory mechanism while mounting a robust IFN response. Strikingly, despite the limited numbers of ISGs in HEK293T cells, we still observed a few ISGs specific for HEK293T cells, including multiple genes (e.g., *IRF7*/*8* and *IFITM2*) with important antiviral and immunoregulatory roles [66,67]. From a functional perspective, we found that the shared ISGs are tightly related to signal transduction and antiviral activity, and likely constitute the core functional layer of the IFN-γ response [7]. As for HeLa-specific ISGs, their function encompasses inflammatory cytokine networks and adaptive immune regulation, thus forming an additional functional layer that expands the shared core module. Although HEK293T-specific ISGs are of a minimal number, they are also associated with the core antiviral effects, suggesting their functional importance. These results corroborate the cell-specificity of the IFN-stimulated transcriptional response and underscore the importance of the cellular context for IFN signaling.

Regarding the cell-specific ISGs as observed in HeLa and HEK293T cells, there are multiple potential underlying mechanisms. First, IFN-γ signaling can induce early and late waves of gene responses, with the early wave dominated by direct STAT1 target genes, while the later wave reflects secondary transcriptional cascades of a broader repertoire of ISGs driven by newly synthesized TFs [7,8]. The overall weaker induction of ISGs in HEK293T cells probably reflects the less efficient propagation of the secondary wave due to lower initial STAT1 activation. In fact, the basal STAT1 expression is substantially higher in HeLa relative to HEK293T cells. Second, several other mechanisms may also account for stronger IFN response in HeLa cells. For example, the IFN-γ receptor *IFNGR1* is strongly induced only in HeLa cells, which may amplify the signaling capacity in HeLa cells during IFN-γ stimulation. Third, gene activation can be influenced by the epigenetic status, including chromatin accessibility, DNA methylation, or histone modifications [68]. Thus, the cell-specific epigenetic patterns may mediate the different induction of many ISGs. Notably, the epigenetic mechanism may explain the HEK293T-specific ISGs that contrast the overall weaker IFN response in HEK293T cells. The specific mechanisms underlying the cell-specific induction of ISGs deserve further studies in the future.

Apart from routine IFN-stimulated genes, this study also characterized the expression of TEs, which are reported to be associated with IFN response by numerous previous studies [36,37,38,42]. Through both subfamily- and locus-level analysis, we concurrently observed a higher degree of TE induction in HeLa relative to HEK293T cells which correlates with the higher level of ISG induction in HeLa cells. Particularly, a total of 17 up-regulated TE subfamilies are identified in HeLa cells, yet not one is in HEK293T cells. Interestingly, the up-regulated TE loci are frequently associated with the identified ISGs and enriched with IFN-related functions, indicating the potential contribution of TEs for regulatory links between these TEs and adjacent ISGs, given that TEs can form interferon-responsive enhancers or promoters according to many previous studies [16,40,41,42]. Moreover, we notice that most of the up-regulated TE subfamilies belong to ERVs, including young primate-specific ones (e.g., HERVP71A-int and HERV4_I-int). Given that some transcribed TEs (particularly ERVs) are capable of forming dsRNAs, which can then induce IFN signaling via “viral mimicry” [38,46], it is also possible that the up-regulated TEs may actively reinforce the IFN response in HeLa cells. Together, these results suggest the potential involvement of TEs to cell-specific IFN responses in HeLa cells. However, our current data only support the correlation between TE activation and ISG induction, and further experiments are necessary to clarify whether and how these TEs regulate the cell-specific induction of ISGs.

We would like to note that this study also has several limitations. First, this study only involves the comparison between two immortalized cell lines. While we uncover some cell-specific features, the conclusions may not be directly generalizable to primary cells or other cell types. Therefore, further studies with more cell types (particularly primary cells) would enable a broader view about the cell-specificity of IFN signaling. Second, this study only includes IFN-γ for investigation, which is the only member of type-II interferon. Given that different groups of IFNs have similar yet not identical roles, further characterization of other types of IFNs would be desirable. Finally, this study only aims at comparing the transcriptomic dynamics across cell types during IFN-γ stimulation, without independent experimental validations (such as by using RT-qPCR) and further pursuing of the underlying regulatory mechanisms. We expect that future studies would be desirable to fully elucidate the mechanisms governing cell-specific IFN responses.

## 5. Conclusions

Overall, this study presents a comparative transcriptomic profiling of IFN-γ responses by using the two widely used human cell lines HeLa and HEK293T as model. We uncover the shared core module and cell-specific patterns of their interferon responses and highlight the potential involvement of TEs for cell-specific ISG induction. Apart from these findings, the RNA-seq data presented in this study may also serve as valuable sources for future studies about IFN responses in human cells.

## Data Availability

All the data generated in this study have been deposited in the NCBI Gene Expression Omnibus (GEO) under accession GSE336141.

## Supplementary Materials

The following supporting information can be downloaded at https://www.mdpi.com/article/10.3390/biology15161418/s1, Figure S1: Hierarchical clustering of RNA-seq samples based on pairwise correlations; Figure S2: PCA plots with the data for HeLa and HEK293T cells analyzed separately; Figure S3: GSEA results for the RNA-seq data during IFN-γ stimulation in HeLa cells; Figure S4: GSEA results for the RNA-seq data during IFN-γ stimulation in HEK293T cells; Figure S5: KEGG enrichment results for the down-regulated genes during IFN-γ stimulation in HeLa cells; Figure S6: Locus-level analysis of TE expression during IFN-γ stimulation in HeLa cells; Figure S7: Locus-level analysis of TE expression during IFN-γ stimulation in HEK293T cells; Figure S8: KEGG enrichment result for the genes adjacent to up-regulated TE loci in HeLa cells; Figure S9: Comparison of the pooled list of DEGs between HeLa and HEK293T cells; Figure S10: Expression profiles of the shared and cell-specific ISGs identified in HeLa and HEK293T cells; Figure S11: Mfuzz clustering of shared ISGs reveals distinct temporal induction patterns in HeLa and HEK293T cells; Figure S12: Expression profiles of representative ISGs for HeLa and HEK293T cells; Table S1: Statistics of the RNA-seq data used in this study; Table S2: Differential gene expression in HeLa and HEK293T cells during IFN-γ stimulation; Table S3: Information of the up-regulated genes associated with up-regulated TEs during IFN-γ stimulation in HeLa cells; Table S4: Comparison of the DEGs identified in HeLa and HEK293T cells.
