# Supplementary material for: Comparative Transcriptomic Profiling in Two Widely Used Human Cell Lines Delineates Their Shared and Distinct Patterns of Interferon Responses
Source: Biology (Basel). 2026 Aug 18;15(16):1418. doi: 10.3390/biology15161418 (PMC13509845; doi:10.3390/biology15161418)
Supplement: Supplementary file 1 [file biology-15-01418-s001.zip › Supplemental Figures.pdf]

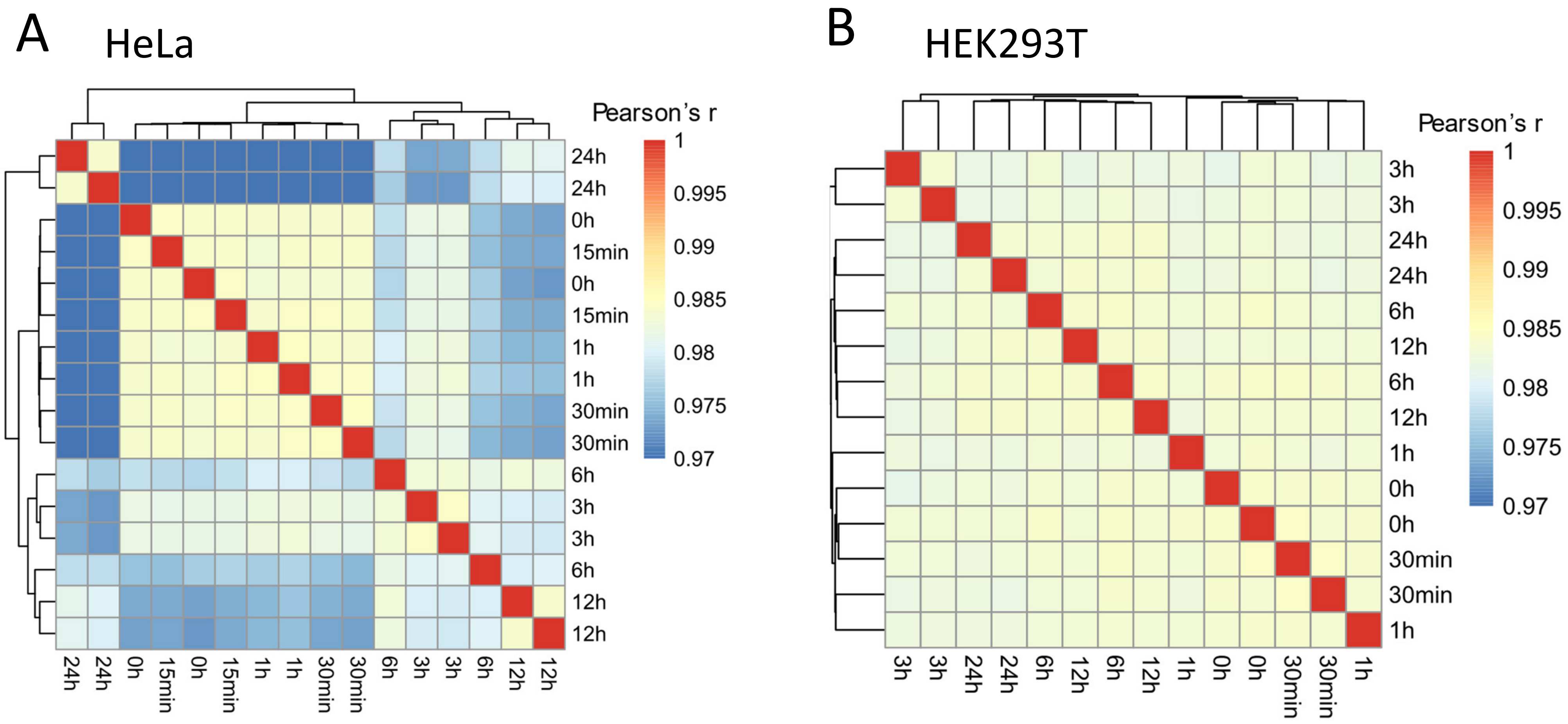

Figure S1 Hierarchical clustering of RNA-seq samples based on pairwise correlation

This figure shows the hierarchical clustering results for HeLa and HEK293T cells respectively, based on pairwise Pearson's r for each cell lines. This figure is related to Figure 1B&C.

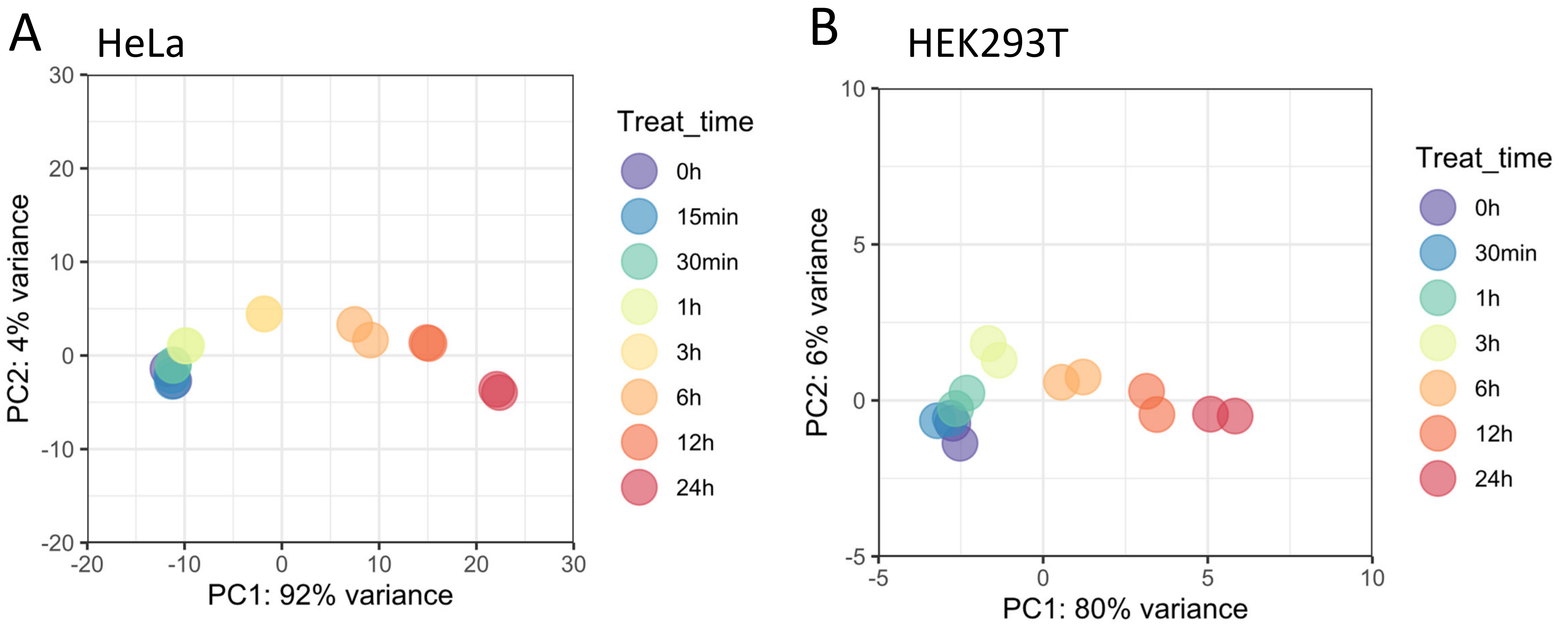

Figure S2 PCA plots with the data for HeLa and HEK293T cells analyzed separately

This figure represents the PCA plots for HeLa (A) and HEK293T cells (B), respectively. This figure is related to Figure 1D, which analyzes and visualizes the data for HeLa and HEK293T cells all together. Given that only less than 100 ISGs are identified in HEK293T cells, here we used the top 100 most variable genes for PCA analyses.

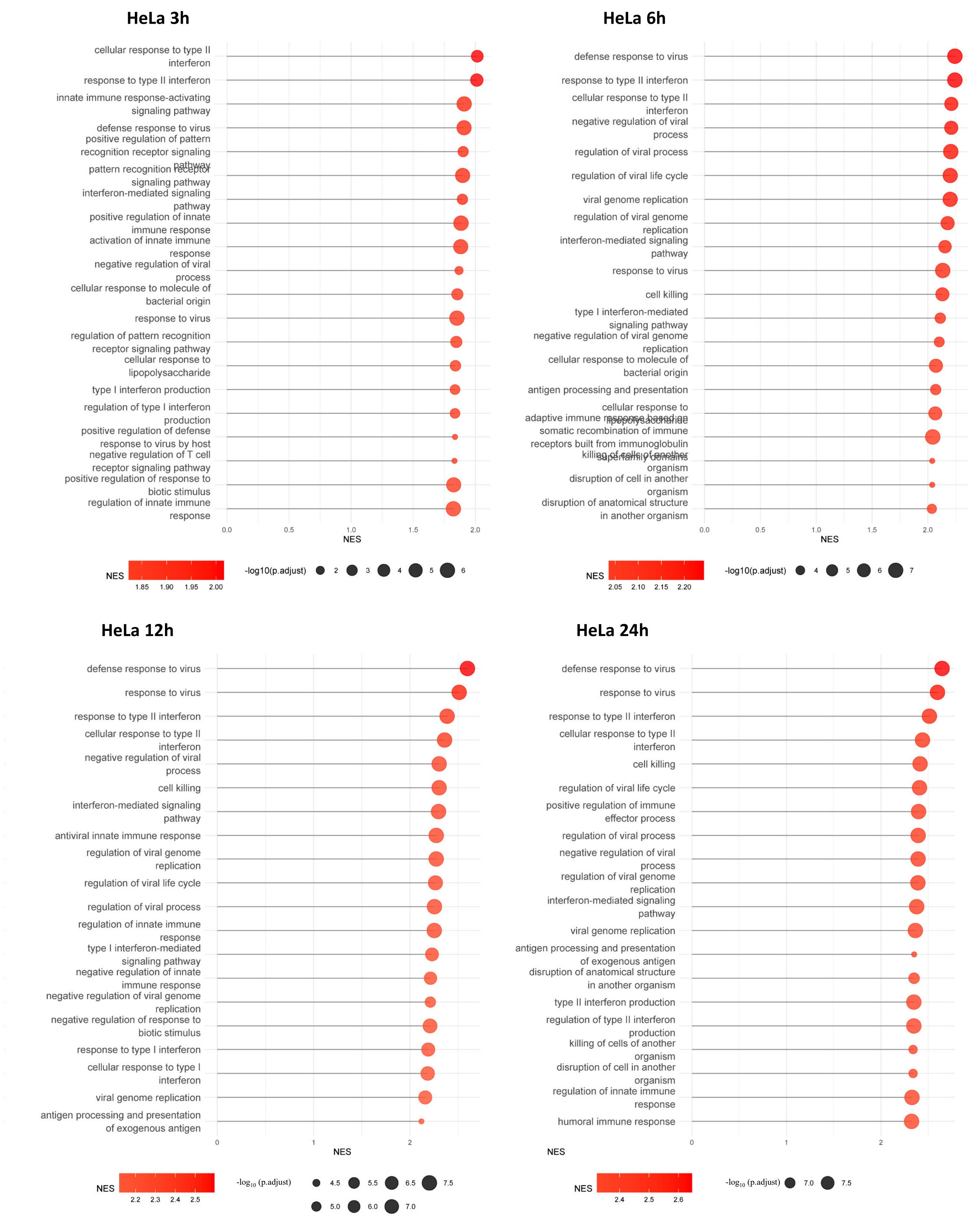

Figure S3 GSEA results for the RNA-seq data during IFN-γ stimulation in HeLa cells

The dot plots show the top enriched biological processes from GSEA analysis at different time points during IFN-γ stimulation in HeLa cells.

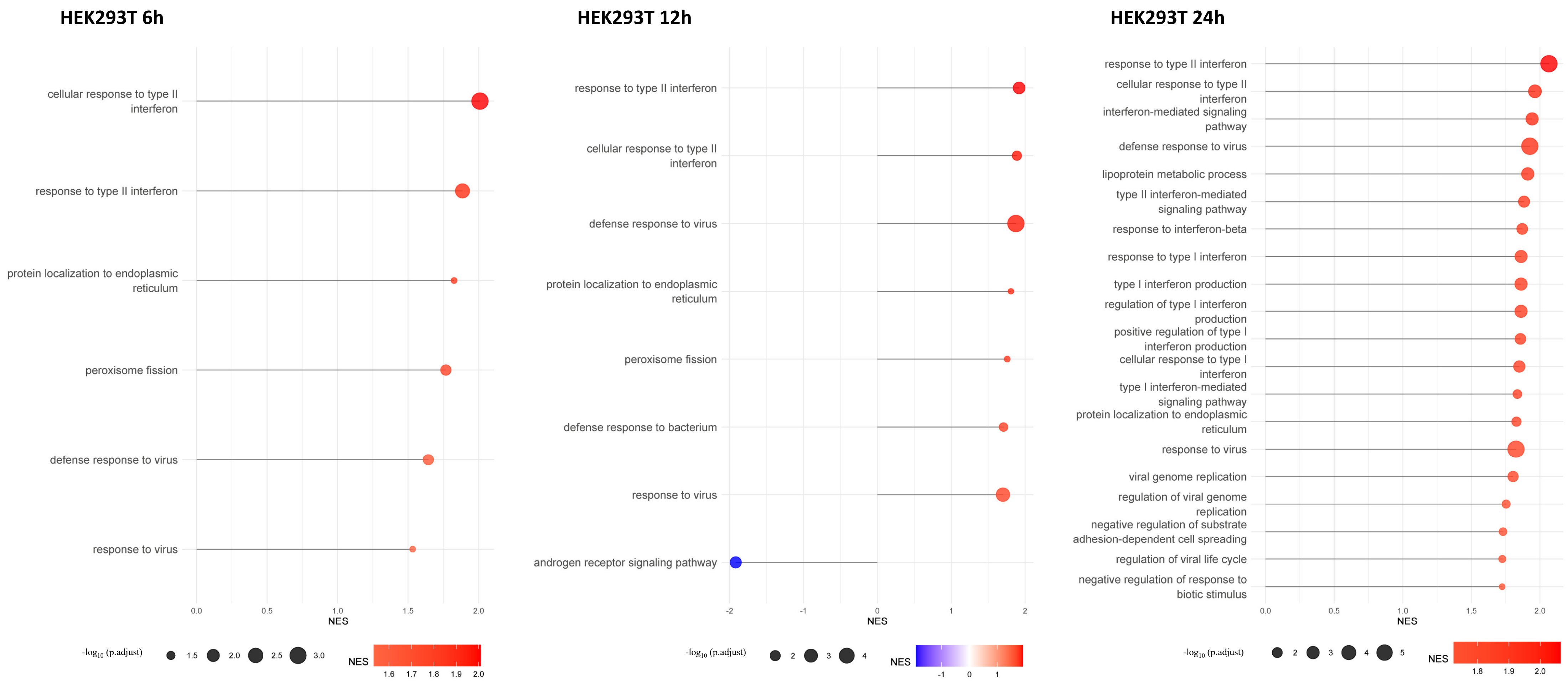

Figure S4 GSEA results for the RNA-seq data during IFN-γ stimulation in HEK293T cells

The dot plots show the top enriched biological processes from GSEA analysis at different time points during IFN-γ stimulation in HEK293T cells.

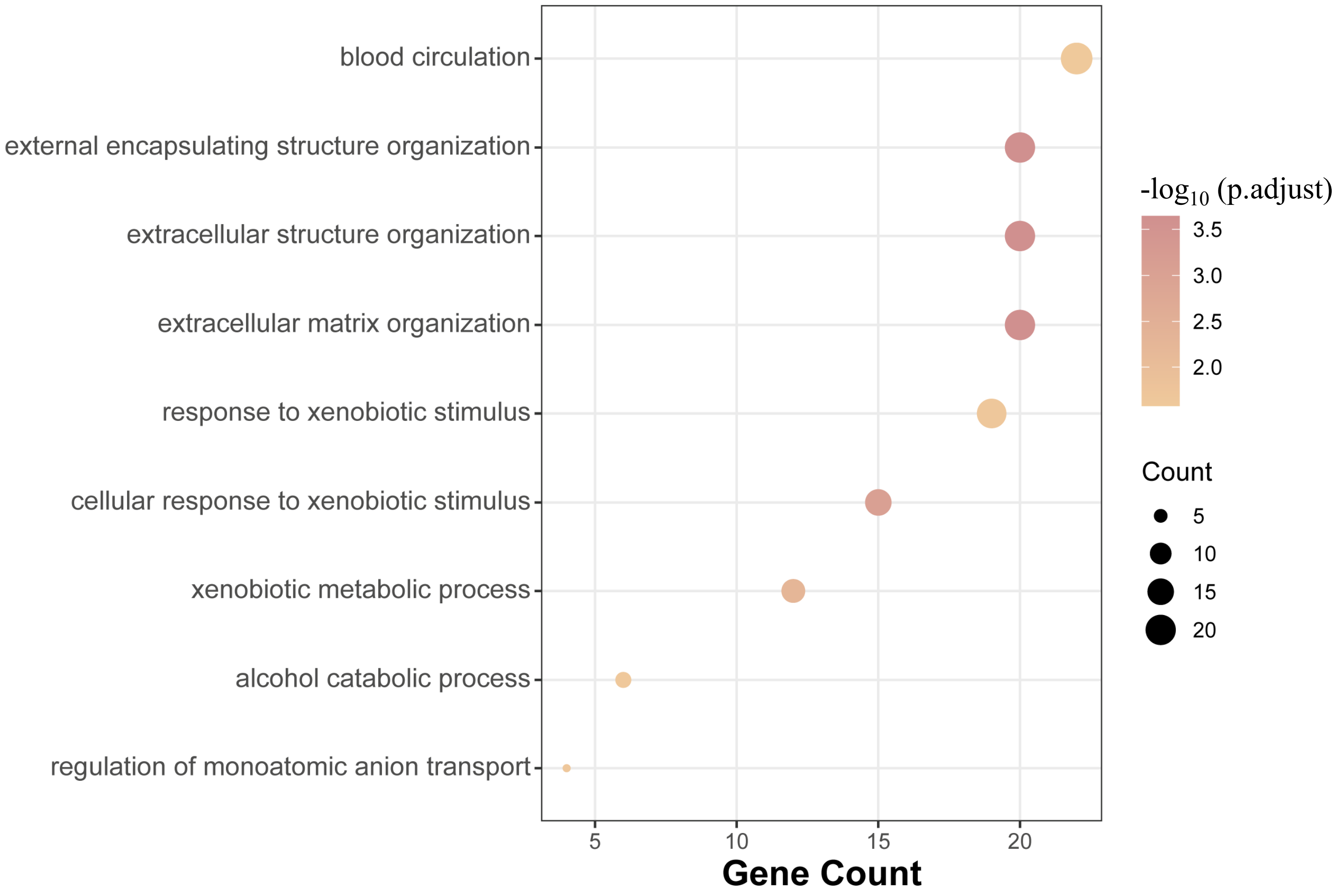

Figure S5 KEGG enrichment results for the down-regulated genes during IFN-γ stimulation in HeLa cells

This figure shows the KEGG enrichment results for all the down-regulated genes at different time points during IFN-γ stimulation in HeLa cells.

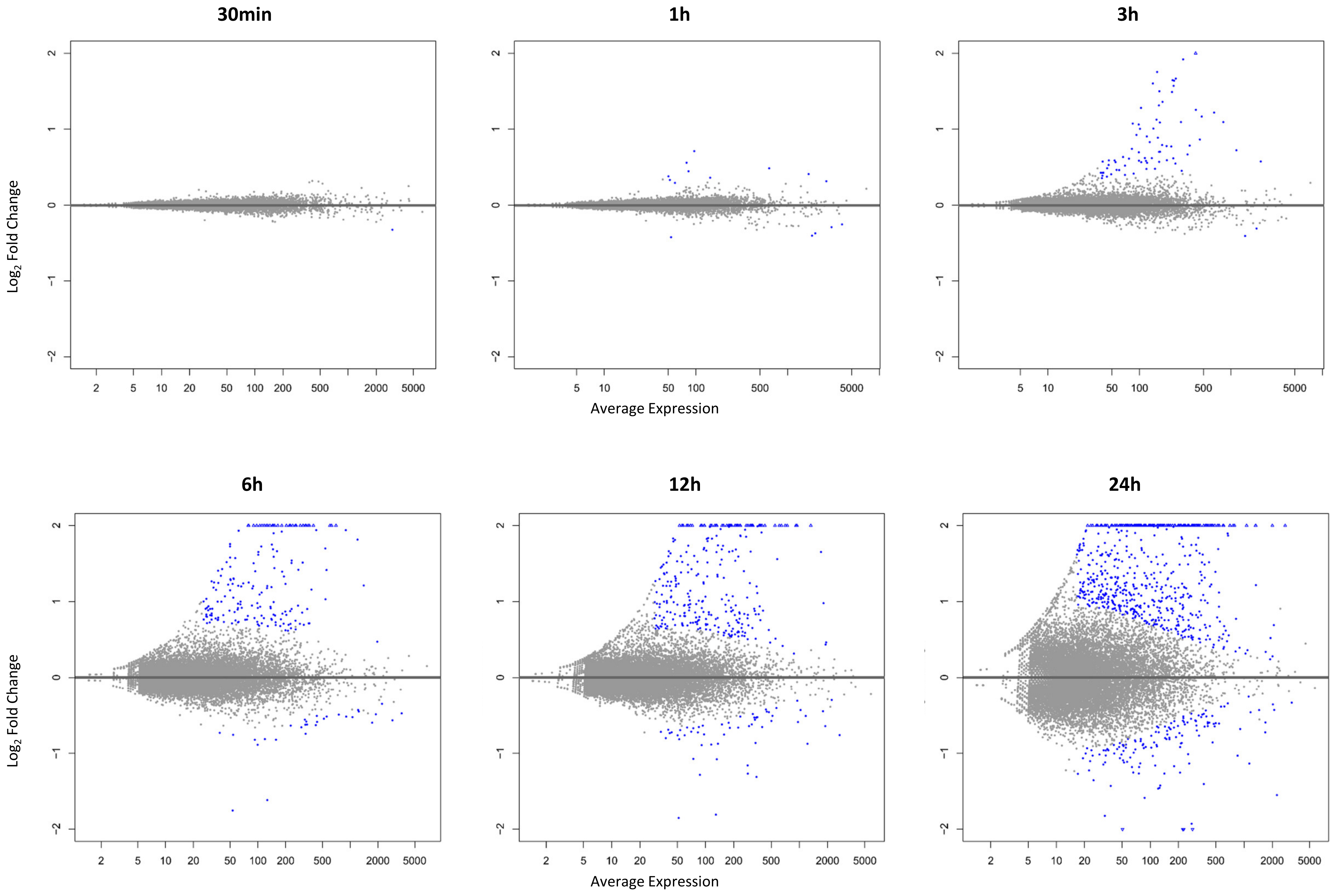

Figure S6 Locus-level analysis of TE expression during IFN-γ stimulation in HeLa cells

MA plots show the expression changes of TE loci at different time points compared to 0 h during IFN-γ stimulation in HeLa cells.

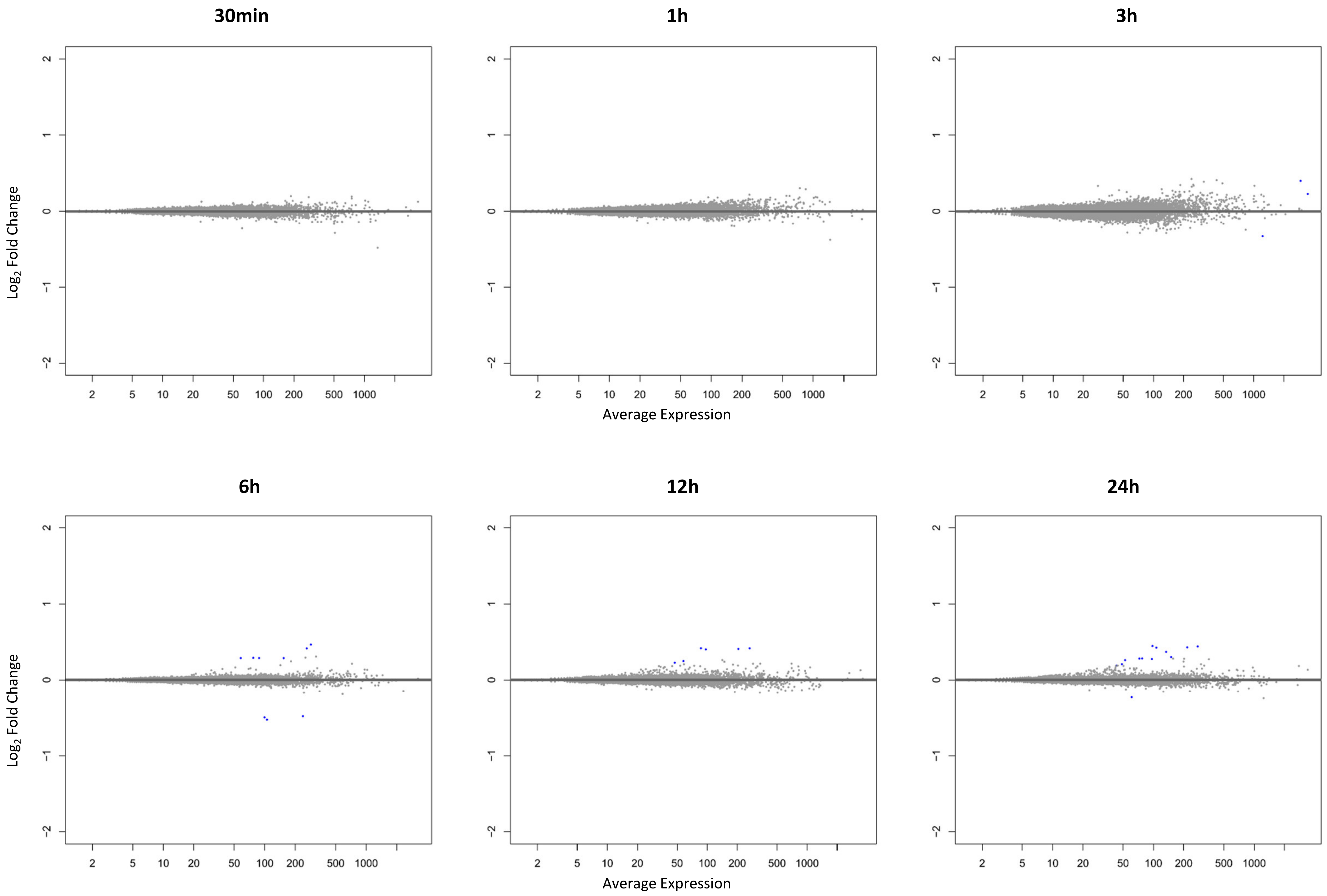

Figure S7 Locus-level analysis of TE expression during IFN-γ stimulation in HEK293T cells

MA plots showi the expression changes of TE loci at different time points compared to 0 h during IFN-γ stimulation in HEK293T cells.

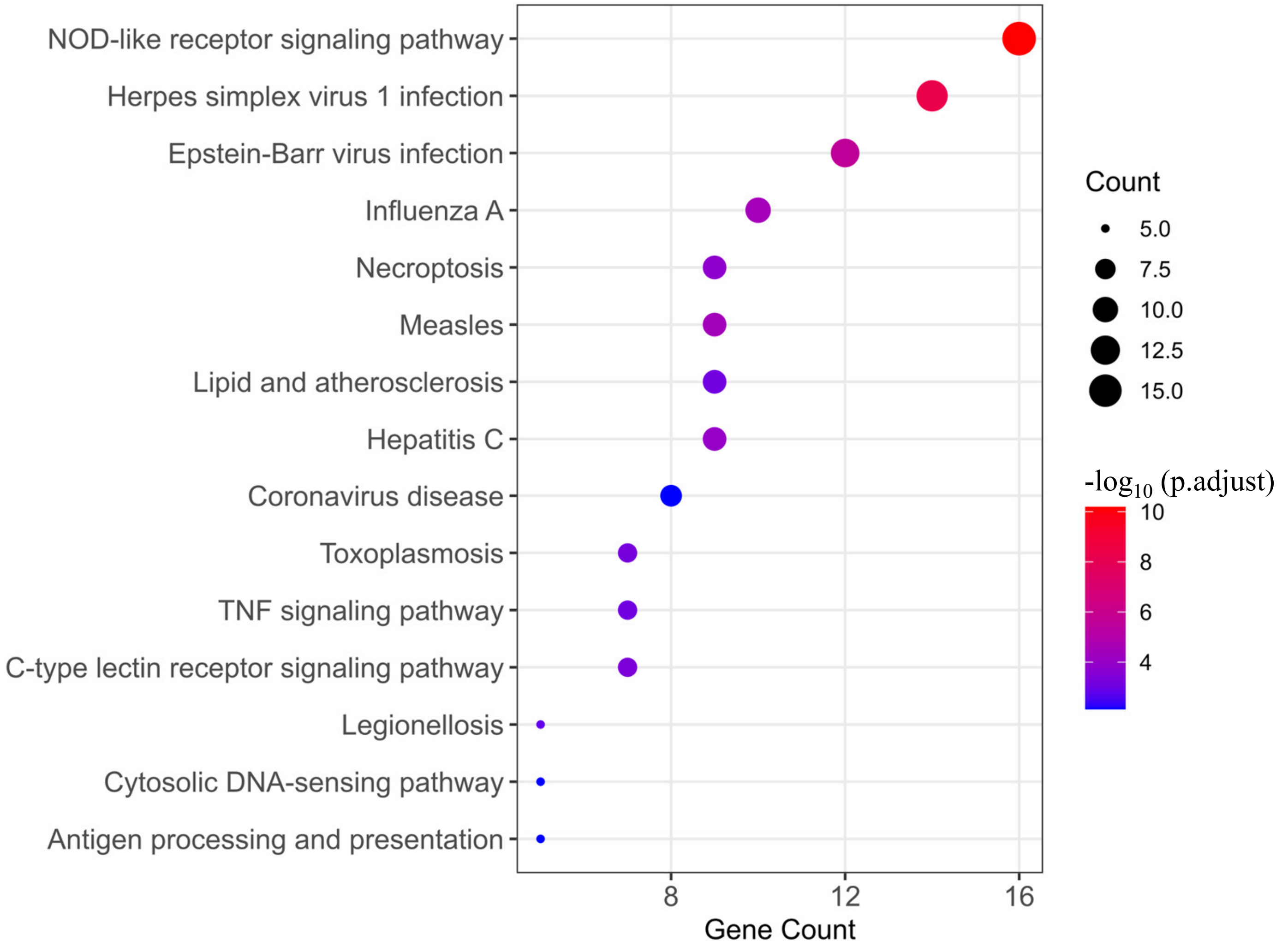

Figure S8 KEGG enrichment result for the genes adjacent to up-regulated TE loci in HeLa cells

This figure shows the KEGG enrichment result for the genes adjacent to the up-regulated TE loci during IFN-γ stimulation in HeLa cells.

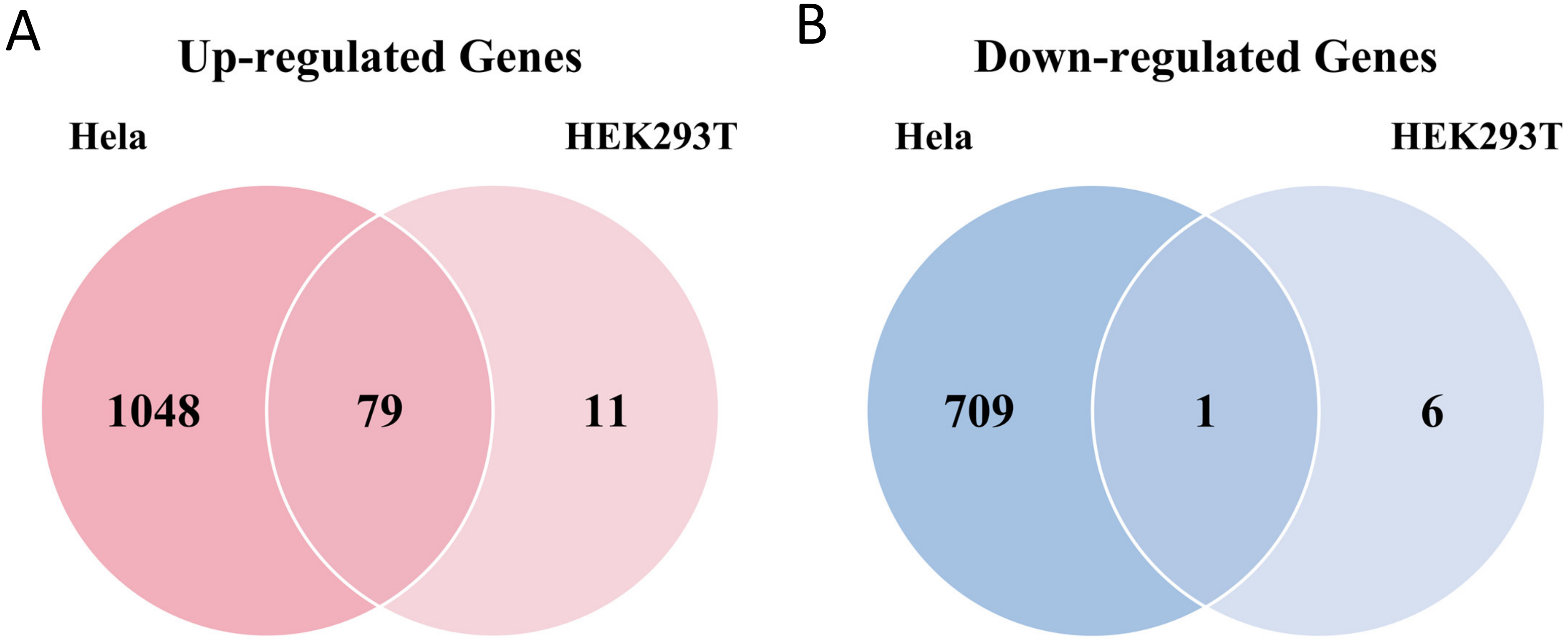

Figure S9 Comparison of the pooled list of DEGs between HeLa and HEK293T cells

The venn plots compare the pooled lists of DEGs during IFN-γ stimulation between HeLa and HEK293T cells. The results for up-regulated (A) and down-regulated DEGs (B) are visualized separately. The ISGs during different time points of IFN-stimulation are pooled to get the unique DEG lists, which are then used for the comparison between cell lines.

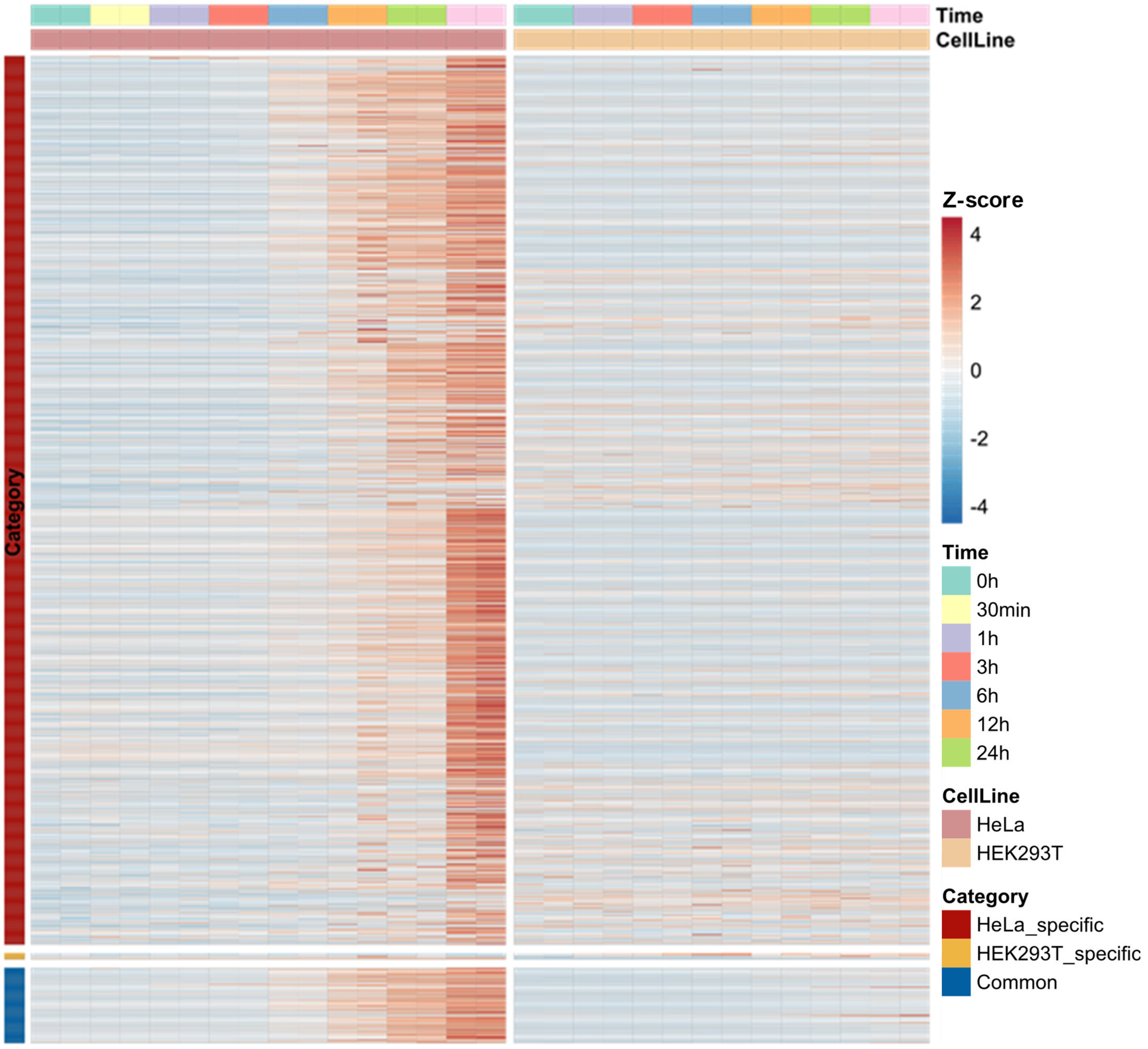

Figure S10 Expression profiles of the common and cell-specific ISGs identified in HeLa and HEK293T cells

The heatmap shows the expression dynamics of common and cell-specific common ISGs in HeLa and HEK293T cells upon IFN-γ stimulation. The color gradients indicated the row Z-score as calculated from normalized read counts. This figure is similar to Figure 4B, except that all the data for HeLa and HEK293T cells are normalized and visualized together.

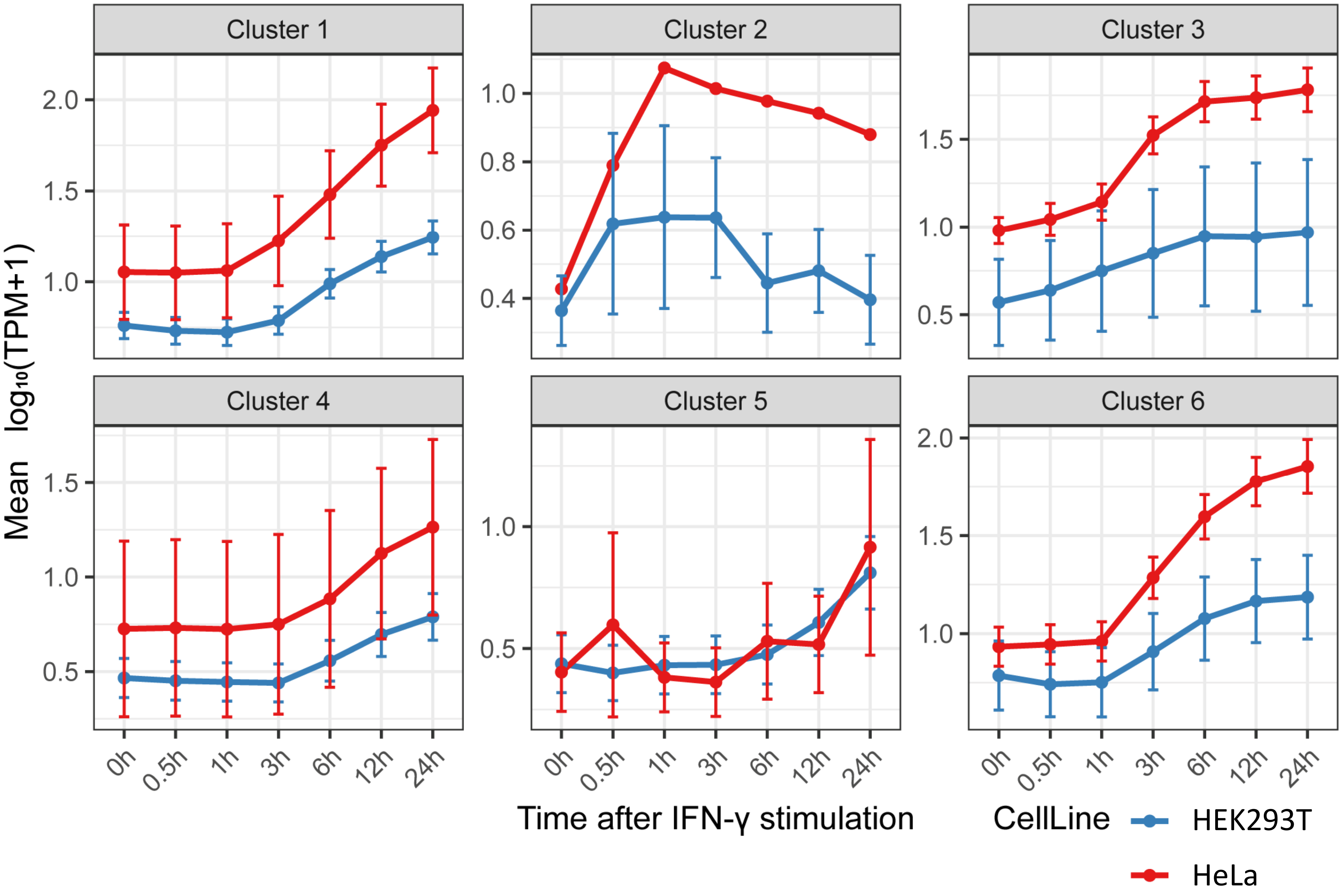

Figure S11 Mfuzz clustering of shared ISGs reveals distinct temporal induction patterns in HeLa and HEK293T cells
Each panel shows the mean expression of shared ISGs assigned to one of six Mfuzz clusters. Red and blue lines represent the profiles for HeLa and HEK293T cells, respectively. Error bars denote ± SEM.

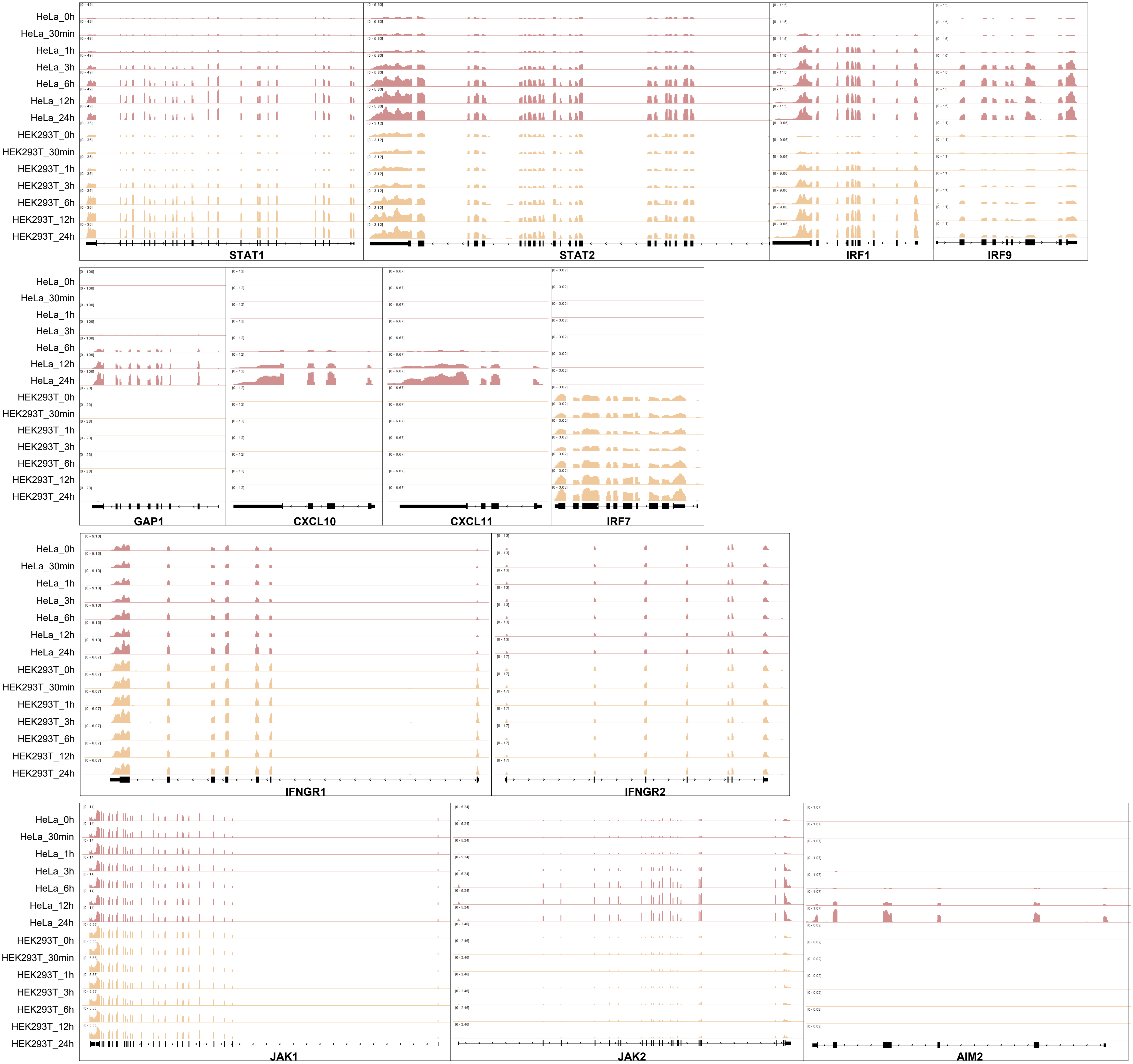

Figure S12 Expression profiles of representative ISGs for HeLa and HEK293T cells

The IGV tracks display the expression profiles of representative common and cell-specific ISGs identified in HeLa and HEK293T cells. Of note, the data for these two cell lines are visualized in their separated scales for better visualization.
